# Intraoperative asymmetry of thalamic stimulation mapping in Essential Tremor: specificity of first hemisphere implantation and transitory lesioning effect

**DOI:** 10.3389/fneur.2026.1893911

**Published:** 2026-10-15

**Authors:** Vittoria Bucciarelli, Dorian Vogel, Jérôme Coste, Rémi Chaix, Simone Hemm, Jean-Jacques Lemaire

**Affiliations:** 1 Institute for Medical Engineering and Medical Informatics, School of Life Sciences, University of Applied Sciences and Arts Northwestern Switzerland, Muttenz, Switzerland; 2 Department of Biomedical Engineering, University of Basel, Allschwil, Switzerland; 3 Université Clermont Auvergne, Clermont Auvergne INP, CHU Clermont-Ferrand, CNRS, Institut Pascal, Clermont-Ferrand, France; 4 Department of Biomedical Engineering, Linköping University, Linköping, Sweden

**Keywords:** deep brain stimulation, essential tremor, hemispheric asymmetry, lesioning effect, probabilistic mapping

## Abstract

**Introduction:**

Deep Brain Stimulation (DBS) effectively reduces symptoms in Essential Tremor (ET). Clinical benefit depends on modulation of distributed neural circuits with still undeciphered mechanisms. Furthermore, the lesioning effect observable intraoperatively following the implantation of the DBS lead in the first brain hemisphere has received little attention. We hypothesize that hemispheric differences exist in the spatial organization of improvement and adverse effects, related to the sequential implantation of electrodes and a transitory lesioning effect.

**Methods:**

We investigated this hemispheric asymmetry with a data-driven, probabilistic mapping approach, linking simulated stimulation volumes [Volumes of Tissue Activated (VTAs)] to symptom improvement (imp) and side effects (se) for each hemisphere. Intraoperative data from 23 ET patients undergoing DBS lead implantation in the ventrointermediate nucleus of thalamus (Vim) were retrospectively analyzed. The left hemisphere was systematically implanted first. Stimulation spots (positions) were categorized using therapeutic window values and side effect occurrence. Hemisphere-specific probabilistic effect volumes of imp (PVimp) and se (PVse) derived from VTAs, and clusters of stimulation spots grouped by category were computed. They were topographically analyzed in relation to structures of the Vim region, and compared within hemispheres and across hemispheres.

**Results:**

In the first implanted hemisphere, PVimp was in the infero-posterior part of Vim reaching the subthalamic tegmental fields, whereas the PVse was above, posterior and lateral to the PVimp, spanning Vim and the ventrocaudal nucleus of thalamus (VC). In the right one, PVimp and PVse overlapped, intersecting Vim and VC, in the intermediate part of both. The stimulation spots clusters, in the left hemisphere, were located around the intermediate part, slightly lateral of Vim and VC to a lesser extent. In the right one, they were located in the intermediate and superior parts of Vim.

**Discussion:**

Significant hemispheric asymmetries were observed, revealing a lesioning effect subsequent to the first DBS lead implantation in the left hemisphere. This led to a broader distribution of optimal positions with a larger PVimp largely overlapping with the PVse in the contralateral right hemisphere. The combined mapping and categorization framework yields hemisphere-specific insights on the transitory lesioning and bi-hemispheric modulation which could guide future DBS strategies.

## 1 Introduction

Chronic Deep Brain Stimulation (DBS) is an established therapy that alleviates symptoms of severe Essential Tremor (ET) (1–3) by modulating neuronal activity within the ventrointermediate nucleus (Vim) of thalamus and neighboring subthalamic region (4–6). The chronic stimulation modulates the dysfunctional network (7) and notably the historic cortico-ponto-cerebello-rubro-thalamo-cortical circuit (8). Although the understanding of the neuronal mechanisms of high frequency DBS is progressing (9, 10), the overall clinical outcome, resulting from the combined modulation of local neuronal populations and distributed brain networks, remains incompletely understood (11). To that end, the understanding of bi-hemispheric modulation is also an issue in ET, notably as ipsilateral effects have been reported (12–14), although not always apparent (15). DBS in general is also associated with a lesioning effect that received little attention. This catch-all word, often called “micro lesioning effect,” encompasses transitory phenomena, hence reversible, linked to intraoperative microelectrode exploration and DBS implantation. The most emblematic one is the improvement of baseline scores up to months, known to be more pronounced if there were more exploratory tracts (14, 16). The degree of improvement observed intraoperatively has been correlated to the clinical outcome (17). Until now, the lesioning effect was only considered as an unspecific, general, phenomenon. Yet intraoperatively, in the second explored hemisphere, clinical effects are linked to the direct stimulation of the second implanted hemisphere, and also to a quasi-experimental lesioning effect following the first explored-implanted hemisphere. To explore this hypothesis, we developed a method to compare size and location of volumes of relevant clinical effects, in the first and second explored-implanted hemispheres. The understanding of these phenomena could shed light on underlying neural correlates of modulation, detailing inter-hemispheric reciprocal influences.

Methodologically, computational modeling of the electrical field enables estimation of the Volume of Tissue Activated (VTA) by specific stimulation settings (18), which can be registered to common anatomical spaces (19–25). This spatial normalization constitutes a critical step enabling group-level statistical analyses across patient cohorts. By combining VTAs with clinical effects, probabilistic mapping allows identification of regions associated with symptom improvement and side effects (26–31). This approach has been used to refine the identification of tremor-suppressing regions such as the posterior subthalamic area (29, 31–35), the Zona Incerta (ZI) (27), and the anterior border of the Vim (30). In such analyses data were pooled from both hemispheres, typically by mirroring one hemisphere onto the other (27, 30, 31, 33, 34). While this increases statistical power, it limits the ability to investigate hemispheric asymmetries in stimulation effects. Here, we hypothesized that hemispheric differences may exist in the spatial organization of improvement and adverse effects, potentially influenced by the sequential implantation of electrodes and associated transient lesioning effects (13, 14, 36). To that end, we retrospectively analyzed intraoperative data from a cohort of ET patients, separating the first implanted (left) and second implanted (right) hemispheres. We combined probabilistic mapping of VTAs with a complementary stimulation spot-based evaluation incorporating therapeutic window (TW) and side effect thresholds. The therapeutic window corresponded to the difference between the minimum current inducing clinical improvement and the threshold at which side effects occur (37). The location of electrode contacts (stimulation spot) strongly influences this balance, giving rise to the concept of a clinical “sweet spot,” where maximal benefit is achieved with minimal adverse effects (38, 39). Probabilistic volumes of improvement (imp) and side effects (se) were identified for each hemisphere independently, and stimulation sites were classified according to clinical criteria into distinct clusters. The stimulation spot clusters and probabilistic volumes were then analyzed in relation to anatomical structures and compared within and across hemispheres. This combined approach provides a refined, clinically relevant, framework for understanding the effect of stimulation, offering new insights into the hemispheric organization of neuromodulation.

## 2 Materials and methods

### 2.1 Clinical material

#### 2.1.1 Patients and surgical procedure

Intraoperative stimulation data of 23 patients (mean age 55.6 ± 19.1 years; 4 females) suffering from severe ET were routinely collected during bilateral Vim-DBS implantation under local anesthesia between 2008 and 2017 at the University Hospital Clermont-Ferrand, France. All the patients signed a written informed consent (Ptolemee Electrophysiologie project: IRB 5921, CE-CIC-GREN-18-03) for the retrospective analysis. The left hemisphere was systematically explored and implanted first and the right about 1 h after. Intraoperative stimulation tests used rigid semi-microelectrodes (Neuroprobe, Sonus; Alpha-Omega Engineering, Israel, contact diameter 0.5 mm, length 1.2–1.5 mm; 130 Hz train, pulse width 60 μs) with average exploration length of 14 mm along two parallel tracks, one central optimized for Vim and Forel H field (F-H-F), and one 2-mm away (secondary) mostly posterior exploring the ventrocaudal nucleus of thalamus (VC) looking for paresthesia. Stimulation spots were tested with 1-mm increments along the trajectories. Systematic early postoperative CT-scan showed absence of brain shift (40). A total of 605 stimulation spots were tested (Table 1), corresponding to an average of 12 sites per hemisphere per patient. Of these, 303 were located on the central trajectory (166 left, 137 right), 254 on the posterior secondary trajectory (138 left, 116 right), 30 on the anterior secondary trajectory (16 left, 14 right), and 18 on the medial secondary trajectory (12 left, 6 right). At each stimulation spot, the current was increased from 0.2 mA to 3 mA in increments of 0.2 mA. For each increment, the effectiveness of symptom improvement was rated by the physician using a 5-point Likert scale (41), converted into linear ratio data: no, 0%; poor, 25%; fair, 50%; good, 75%; excellent, 100%. In total, 696 stimulation tests were recorded. Among these, 139 tests (≈23%) elicited side effects, with 64 observed in the left hemisphere and 75 in the right. Reported side effects included paresthesia (*n* = 91), dysarthria (*n* = 26), oculomotor disturbances (*n* = 11), vegetative symptoms (*n* = 6), and pyramidal signs (*n* = 5).

**Table 1:** Intraoperative stimulation spots (*n* = 605), weighted mean percentage of improvement, and side effects by hemisphere for the 23 ET patients.

Stimulation spots	Left hemisphere	Right hemisphere
N [average/patient]	332 [14]	273 [12]
Weighted mean improvement [median; min; max] [%]	54.3, [50; 0; 100]^***^	65.2 [75; 0; 100]^***^
side effects (*n*) [%]	Par: 35 [10.5%]^*^	Par: 56 [20.5%]^*^
	Dys: 14 [4.2%]	Dys: 12 [4.4%]
	Ocu: 9 [2.7%]	Ocu: 2 [0.7 %]
	Veg: 5 [1.5%]	Veg: 1 [0.37%]
	Pyr: 1 [0.3%]	Pyr: 4 [1.5%]

The improvement was weighted by the frequency with which each score appeared in the dataset. Par, paresthesia; Dys, dysarthria; Ocu, oculomotor disturbances; Veg, vegetative symptoms; Pyr, pyramidal signs. The asterisks indicate statistically significant differences calculated with the Mann–Whitney U-test for improvement values and with Fisher's exact test for side effect occurrence: ^*^p < 0.05; ^***^p < 0.001.

#### 2.1.2 Stimulation spot categories

Six categories of stimulation spots were specified according to their TW and occurrence of side effects (Table 2) (29, 42). Categories 1a and 1b were characterized by a TW equal to 0 mA and were classified as clinically unusable sites, as the stimulation amplitude required to induce clinical improvement was not reached. In category 1a, stimulation immediately induced side effects, whereas in category 1b it produced neither clinical improvement nor side effects. Categories 2a, 2b, 2c, and 3 were characterized by a TW > 0 mA and were classified as clinically effective sites. To further differentiate stimulation selectivity, TW values were categorized as small or large using a threshold of 1.5 mA, based on the observed range (0–3.0 mA). Furthermore, the presence or absence of side effects at the upper stimulation limit was taken into account. Categories 2a and 2b were associated with side effects and corresponded to small and large TWs, respectively. Categories 2c and 3 were not associated with side effects and likewise corresponded to small and large TWs, respectively. Among these, category 3 was considered as the most optimal and representative of the clinical sweet spot.

**Table 2:** Summary of stimulation spot category characteristics and number of tested stimulation spots per category.

Clinical applicability	Category	TW [mA]	se [Yes/No]	Stimulation spots [Total (Left/right)]
Irrelevant	**1a** 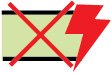	0	Yes	50 (17L; 33R)
	**1b** 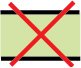	0	No	58 (38L; 20 R)
Clinically effective	**2a** 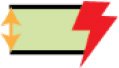	< 1.5	Yes	66 (42L; 24R)
	**2b** 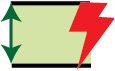	≥1.5	Yes	23 (5L: 18R)
	**2c** 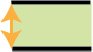	< 1.5	No	244 (155L; 89R)
Optimal (clinical sweet spot)	**3** 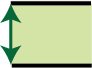	≥1.5	No	164 (75L; 89R)

The numbers in the “Stimulation spots” column are reported in the following format: total (left hemisphere; right hemisphere). TW, therapeutic window; se, side effects; L, left; R, right.

### 2.2 Probabilistic mapping

The steps applied to generate probabilistic stimulation maps are detailed in Hemm-Ode et al. (43) and Bucciarelli et al. (44) and in the Supplementary material – Section 1. The software ELMA^1^ (45) was used to generate patient-specific brain conductivity models in which tissue classes of voxels (cerebrospinal fluid, 2 S/m; gray matter, 0.123 S/m; white matter 0.075 S/m) were derived from each individual anatomical MRI. The models incorporated heterogeneous but isotropic tissue conductivities. Electric Field (EF) simulations were run in Comsol Multiphysics 5.5 (COMSOL AB, Sweden) for each patient and each tested stimulation setting. The EF meshes were thresholded at 0.2 V/mm (46, 47) to obtain the VTAs. 696 VTAs (381 left hemisphere + 315 right hemisphere) were computed (Figure 1A). The individual VTAs, labeled with the respective DBS effect, were transformed using a non-linear approach based on the pre-operative T1, to a group-specific MRI template (AC-PC aligned) (24), enabling group analysis. The VTAs associated with improvement scores were used to compute the Probabilistic Volume of Improvement (PVimp). The voxels with significant effectiveness were selected using a Bayesian one-sample *t*-test (44, 48) with null hypothesis H0: improvement < 75% and alternative hypothesis: H1: improvement ≥ 75%. The Bayesian *t*-test was selected based on our previous methodological studies (44, 49, 50), in which it was benchmarked against conventional non-parametric approaches (e.g., Wilcoxon test). Those studies demonstrated greater robustness to outliers and more stable statistical inference, while a sensitivity analysis confirmed that the results were robust to the choice of prior. As the probabilistic volumes analyzed in the present study were previously validated on the same cohort using a leave-one-patient-out strategy (44), no additional cross-validation analyses were performed in the present work. The converted linear ratio of 75% was used as a clinically relevant improvement threshold (51). The threshold of statistical significance was fixed at 10 (Bayes Factor, ranging from 0 to infinity) (52). This Bayes Factor value is comparable to a *p-value* of 0.01 (53). Given the low number of VTAs associated with side effects, all the side effect types were grouped together to obtain a general Probabilistic Volume of Side Effects (PVse). The information on side effects was binary (side effects / no side effects). Therefore, each voxel was tested with a binomial test. This approach, used also in Dembek et al. (54), was chosen because it directly estimates the voxel-wise probability of observing a side effect, providing an inferential statistical framework for binary clinical outcomes. The *p-values* were corrected for multiple comparisons with False Discovery Rate (55) and the voxels with *p* < 0.05 were retained as part of the PVse. For both PVimp and PVse, two occurrence parameters restricted the selected voxels (35, 44): the patient frequency, set at 25%, thus at least 6 patients must share the selected voxel; the VTA frequency, set at 10% of the maximum number of VTAs encompassing a voxel. These threshold values were chosen for consistency with previous studies (35, 44), although they remain inherently arbitrary. To assess the robustness of our findings, a sensitivity analysis has been conducted in another study using alternative threshold values (49). Given that the primary objective of this study is the comparison between hemispheres, the critical requirement was the consistent application of identical thresholds to both sides. Spread, small volumes of voxels ≤ 1 mm^3^ were excluded from PVimp and PVse, since their selection was likely to occur by chance due to random noise (56, 57) (Figure 1B).

**Figure 1:**
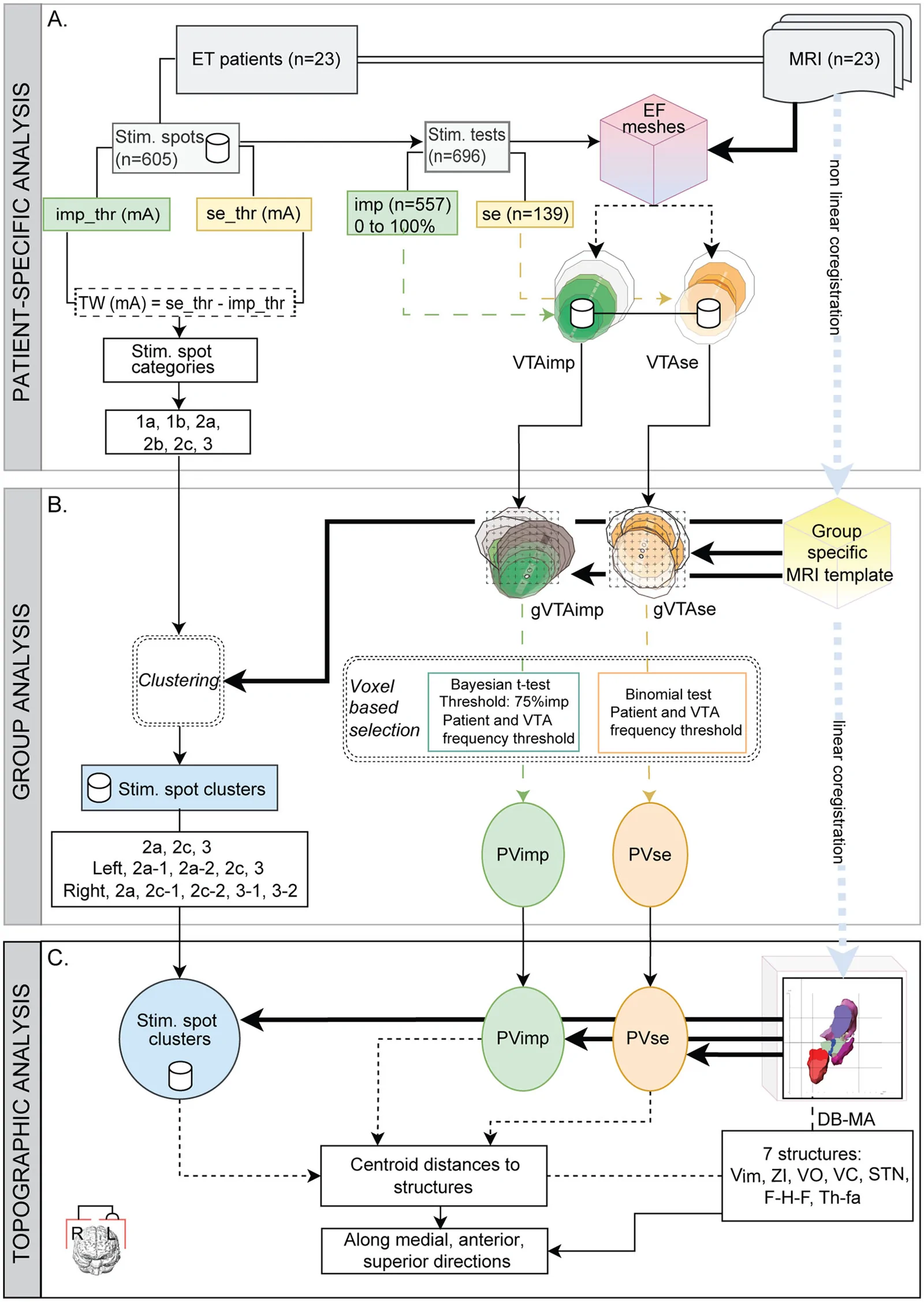
Overall workflow. **(A)** Collected stimulation data: stimulation spots and related stimulation tests allowing for the simulation of the Volumes of Tissue Activated. Each stimulation spot was categorized according to its therapeutic window and occurrence of side effects and VTAs were labeled with respective effects (imp, improvement / se, side effects). **(B)** Group analysis in cohort-specific anatomical template with voxel-wise statistical testing and clustering of stimulation spots. **(C)** Topographic analysis of stimulation spot clusters and probabilistic volumes according to DB-MA (58) structures. ET, Essential Tremor; Stim, stimulation; imp, improvement; se, side effects; TW, therapeutic window; thr, threshold; EF, Electric Field; VTA, Volume of Tissue Activated; gVTA, grouped VTAs; Vim, ventrointermediate nucleus of thalamus; ZI, zona incerta; VO, ventrooral nucleus of thalamus; VC, ventrocaudal nucleus of the thalamus; STN, subthalamic nucleus; F-H-F, Forel's field H; Th-fa, thalamic fascicle.

### 2.3 Stimulation spots clustering

Density-based spatial clustering of applications with noise (DBSCAN) (59) was applied to assess whether stimulation spots belonging to the same category would cluster in a specific location in the group-specific MRI template. A maximum distance of 1.5 mm was used to define neighboring stimulation spots within a cluster. This choice reflects the 1 mm spacing between consecutive stimulation spots along the trajectory, while allowing for minor spatial variability. The minimum cluster size was set to 5 stimulation spots to exclude isolated or sparsely distributed points. Only 317 stimulation spots of the categories 2a (*n* = 21), 2c (*n* = 196), and 3 (*n* = 100) formed clusters. Five clusters were obtained in the right hemisphere (2a, 2c-1, 2c-2, 3-1, 3-2) and four in the left hemisphere (2a-1, 2a-2, 2c, 3); the indices of subclusters were arbitrarily attributed, 1 for superior, and 2 for inferior (Figure 1B).

### 2.4 Intra- and inter-hemispheric topographic analysis

The topographic, anatomic, analysis was realized using the Deep Brain–MRI Architecture (DB-MA) atlas (58). The group-specific MRI brain template was registered to the DB-MA atlas space [rigid and affine transforms; BRAINS module; 3D Slicer; Boston, USA (60)]. This enabled visualization of the Vim region, including fiber systems. Seven structures were selected for analysis: the Vim, the ZI, the ventrooral nucleus of thalamus (VO), the ventrocaudal nucleus of the thalamus (VC), the subthalamic nucleus (STN), and two Forel's fields, the field H that contains the prelemniscal radiations (F-H-F) and the field H1 or thalamic fascicle (TH-fa) (Figure 1C). PVimp and PVse, as well as stimulation spot clusters, were analyzed in relation to these structures. Their volumes and spatial distributions were compared both within (intra-hemispheric) and across (inter-hemispheric) hemispheres. Centroid locations of PVimp, PVse, and stimulation spot clusters were calculated with respect to the above-mentioned anatomical structures along the medial–lateral, anterior–posterior, and superior–inferior axes. Only distances relative to Vim were considered for statistical analysis. Intra- and inter-hemispheric comparisons of the positions of the points belonging to stimulation spot clusters were performed using multivariate ANOVA with *post hoc* Tukey HSD tests. The assumptions underlying multivariate ANOVA were assessed by evaluating univariate normality using Shapiro–Wilk tests and multivariate outliers using Mahalanobis distance. Intra-hemispheric analyses aimed to determine whether stimulation spots from different clinical categories clustered in distinct anatomical locations within each hemisphere. Accordingly, clusters were compared across clinical categories separately for each hemisphere. Inter-hemispheric analyses aimed to assess whether stimulation spots belonging to the same clinical category differed in location between hemispheres. Thus, clusters of the same clinical category were compared between hemispheres. These comparisons were conducted by analyzing subclusters separately. All analyses were performed in Python.

## 3 Results

### 3.1 Volumes

The volume of PVimp in the right hemisphere (457 mm^3^) was larger (about 12 times) than that of the left (37 mm^3^) PVimp. For PVse the opposite trend was observed to a lesser extent (left PVse: 388 mm^3^; right PVse: 285 mm^3^, about 73%). The volumes of stimulation spot clusters were smaller in the right hemisphere for all the stimulation spot categories (Table 3).

**Table 3:** Volumes of identified stimulation spot clusters in the right and left hemispheres.

Stimulation spot clusters	Volume [mm^3^]	Hemisphere
2a	0.1	Right
2a-1	1.61	Left
2a-2	0.51	Left
2c-1	42.4	Right
2c-2	0.8	Right
2c	186.7	Left
3-1	1.62	Right
3-2	41.3	Right
3	57.5	Left

### 3.2 Topography

The topography of PVimp, PVse and the identified stimulation spots is shown in Figure 2 and Supplementary Figure S1. The topography of the stimulation spots clusters is presented in Figure 3. The inter-centroid distances between PVs and stim-state clusters, and the 7 structures of the Vim region are summarized in Supplementary Tables S1, S2. Globally, PVimp extended medially and inferiorly to PVse. PVimp and PVse were located in the posterior Vim region, PVse being the most posterior. In the left hemisphere, PVimp was more anterior, medial, and inferior, with respect to the PVimp in the right hemisphere. The left PVse was more posterior, lateral and inferior to the right PVse.

**Figure 2:**
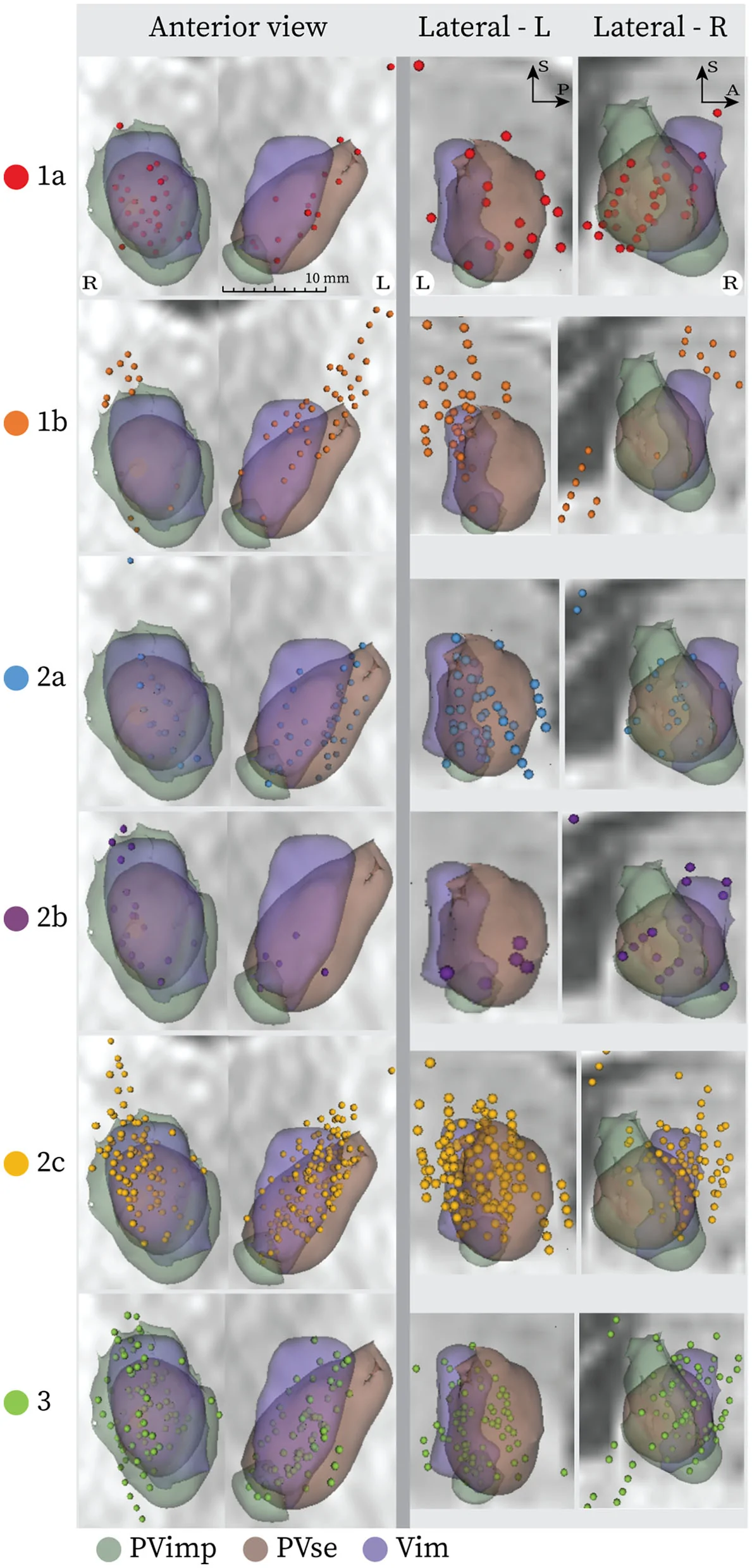
Topography of stimulation spots (dots) according to clinical categories (from top to bottom, 1a, red; 1b, dark orange; 2a, blue; 2b, purple; 2c, pale orange; 3, green), and of probabilistic effect volumes (PVimp, green; PVse, brown); the Vim (light purple) is displayed; right, R, and left, L, hemispheres; left column, anterior view, right column, lateral views.

**Figure 3:**
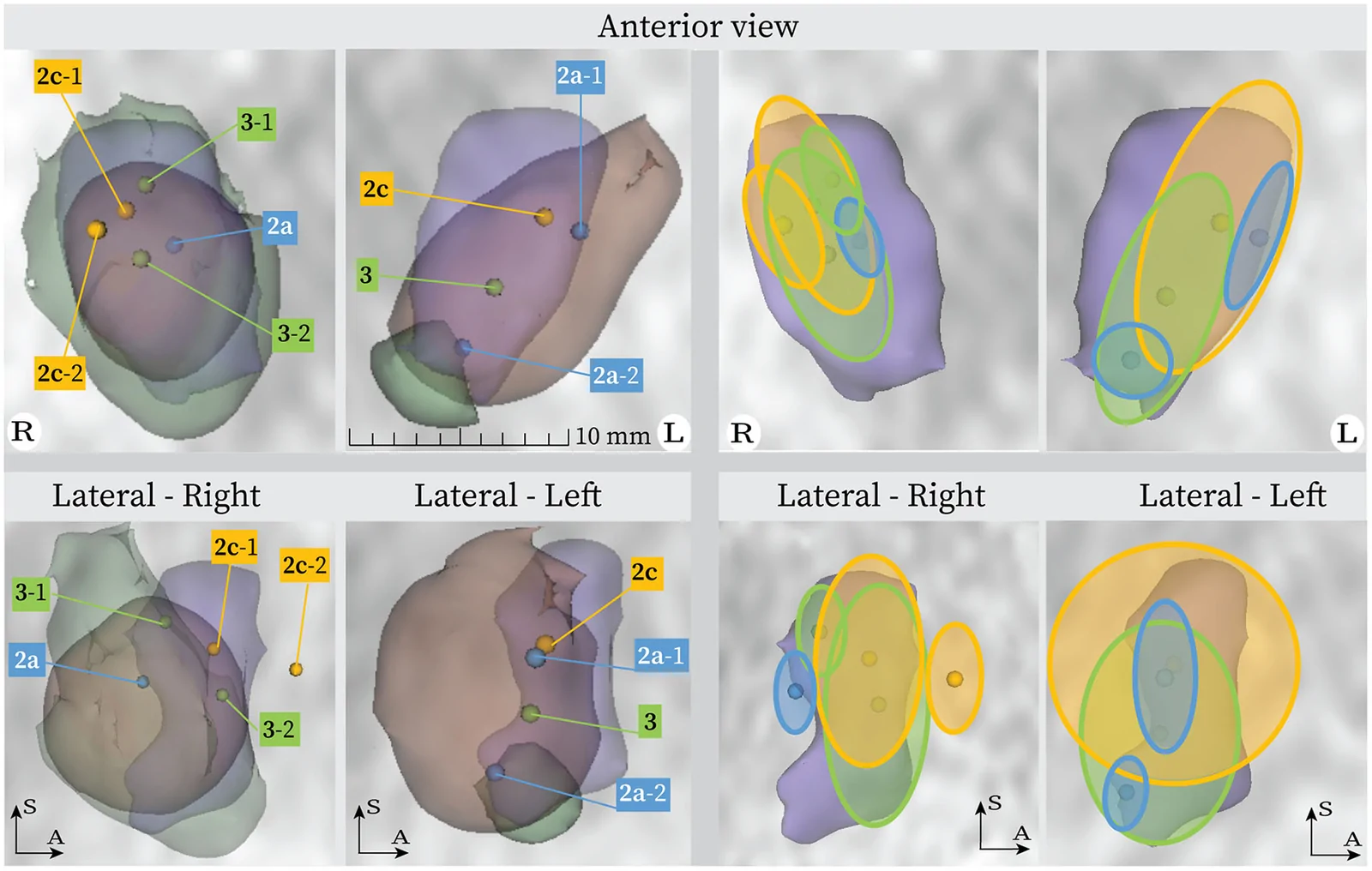
Topography of stimulation spot clusters, 2a, 2a-1, 2a-2, 2c, 2c-1, 2c-2 and 3, 3-1, 3-2; probabilistic effect volumes (PVimp, green; PVse, brown) and the Vim (light purple) are displayed; The anterior and lateral views in the first 2 columns on the left show the PVimp, PVse, Vim and centroids of the clusters. The 2 columns on the right show Vim and cluster centroids and volumetric extent.

Stimulation spot clusters, considering the divisions in subclusters, had significantly different locations within the left and right hemispheres and between hemispheres (multivariate ANOVA *p* < 0.05, Figure 4). In the left hemisphere, cluster 3 was medial and inferior to {2c, 2a-1}, and 2a-1 was superior, anterior and lateral to 2a-2 (Figure 4A). In the right hemisphere, 3-2 was medial to 2c-1, anterior to {3-1, 2a}, and inferior to {3-1, 2c-1}. 3-1 was also posterior to {2c-1, 2c-2}, 2c-2 was the most anterior, 2a- was the most posterior (except 3-1) (Figure 4B). Between the right and left hemispheres: left {3, 2c, 2a-2} were more medial, respectively than right 3-1 & 2, 2c-1 and 2a, but left {2a-1} was less medial than right 2a; right {3-2, 2c-2, 2a} were more anterior, respectively than left 3, 2c and 2a-2; and right {3-1, 2a} were more superior, respectively than left 3 and 2a-2 (Figure 4C).

**Figure 4:**
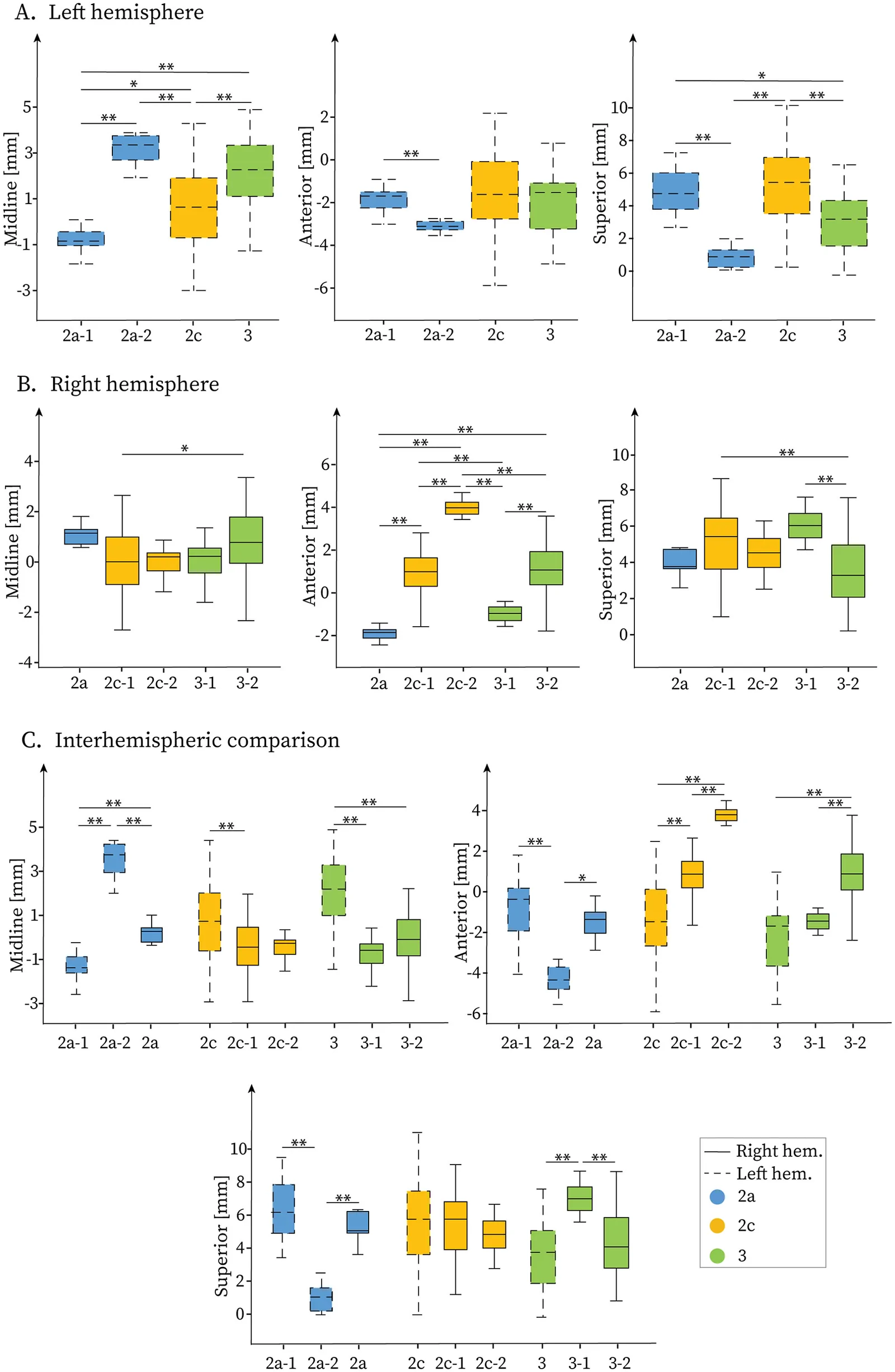
Distributions of the positions (mm) of stimulation spots included in clusters (2a: blue; 2c: yellow; 3: green), by hemisphere [**(A)** left, dotted line; **(B)** right, solid line] and between hemispheres **(C)**. The positions of the stimulation spots were calculated with respect to Vim's centroid. The boxplots show median and interquartile range. Left column: transversal orientation, positive toward medial. Intermediate column: longitudinal orientation, positive toward anterior. Right column: inferior-superior orientation, positive toward superior. Statistically significant differences: *p < 0.05, **p < 0.01.

The topography of PVimp, PVse and stimulation spot clusters in the Vim region according to DB-MA is in Figure 5. This revealed, in the left hemisphere, the overlaps of PVimp with the inferior Vim and the rubro-thalamo-cortical fibers, whereas, in the right hemisphere, PVimp and PVse overlapped in the posterior Vim, crossing two fiber systems, rubro-thalamo-cortical and thalamo-cortical, in the intermediate part of Vim.

**Figure 5:**
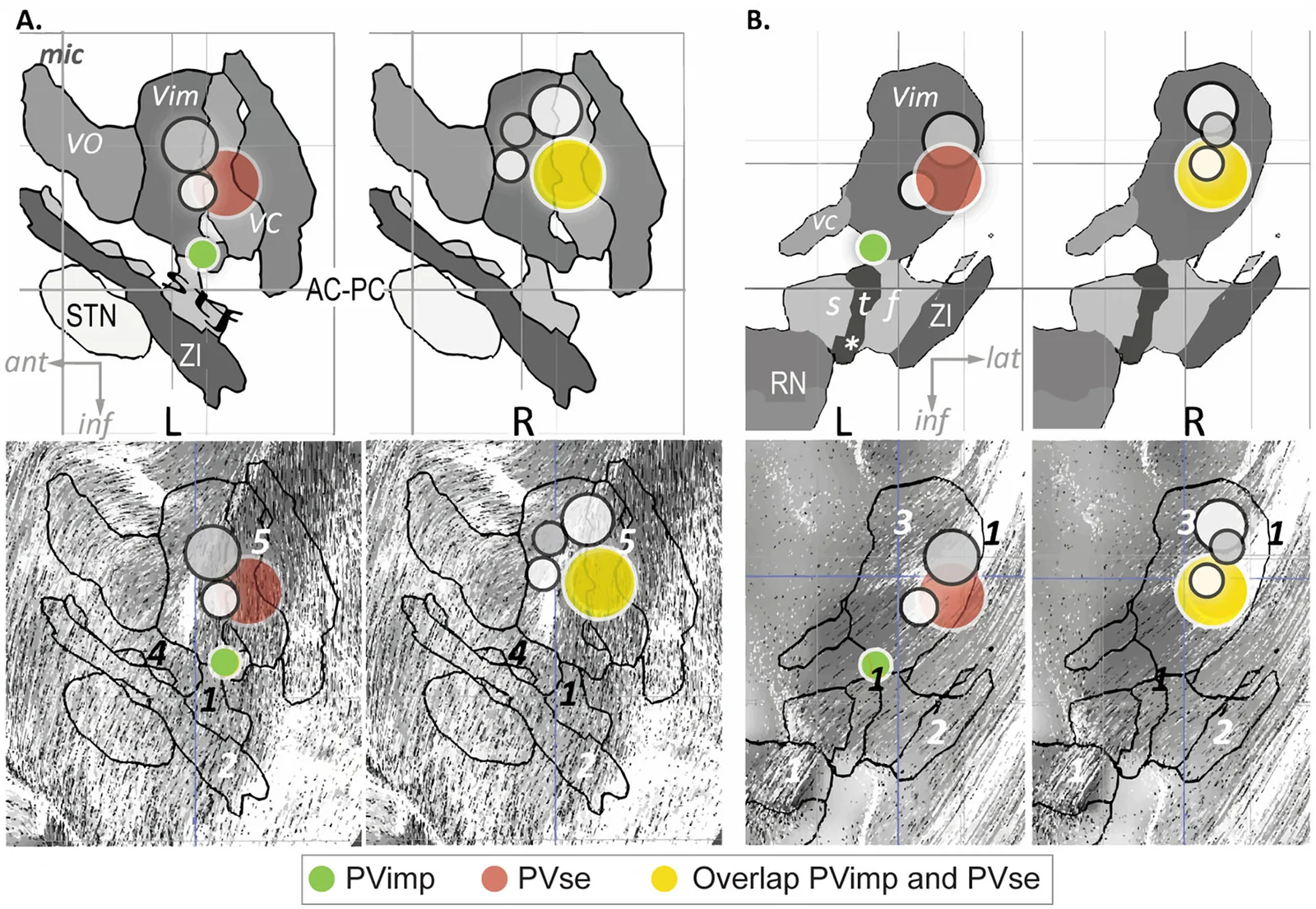
Topography of probabilistic volumes (PVimp, green; PVse, red; yellow when they overlap) and stimulation spots clusters (gray, only clusters >1.5 mm^3^ are displayed: left 3 and 2-c; right 3-1, 3-2, and 2c-1) after registration to the DB-MA atlas (Supplementary Figure S2): **(A)** lateral, and **(B)** anterior, views of sections going through the centre of Vim (AC-PC alignment; mic, intercommissural coronal plane); top rows, structures, bottom rows, fiber systems (1-mm thickness; fiber tracings in gray); RN, red nucleus, stf, subthalamic tegmental fields, *F-H-F, (1) rubro-thalamo-cortical fibers, (2) trans-zona-cortico-subthalamic fibers, (3) thalamo-cortical fibers, (4) subthalamo-cortical fibers, (5) VC-cortical fibers (see text for abbreviations).

## 4 Discussion

We identified tremor-related PVimp and PVse, and categorized stimulation spots into clinically relevant categories, combining probabilistic mapping with stimulation spot clustering. We found pronounced inter-hemispheric differences in the topography of the PVs, indicating a transitory anti-tremor lesioning effect.

### 4.1 Tremor-related analysis

Regarding probabilistic volumes, in the first implanted (left) hemisphere, PVimp was located in the infero-posterior part of Vim, reaching the subthalamic tegmental fields traversed by rubro-thalamo-cortical fibers. In contrast, PVse was positioned more superiorly, posteriorly, and laterally, spanning Vim and VC. In the second implanted (right) hemisphere, PVimp and PVse overlapped within the intermediate part of Vim and VC. These locations of effects are consistent with DBS mapping and clinical studies in ET (1–3, 29, 32–34, 61, 62), including the relationship between VC and paresthesia (42). Also, most of the stimulation spots fell within the clinically relevant clusters (Figures 2 and 3), reflecting accurate targeting of anti-tremor region. Optimal stimulation spot positions (category 3) were generally located closer to PVimp, with respect to category 2c sites, and showed a more medial and inferior distribution. Category 2a sites, much less frequent than 2c, were also closer to PVimp but not statistically significant. The higher proportion of category 2c spots in the left hemisphere (Table 2) may reflect the small size of the optimal area (PVimp), such that a higher number of stimulation spots located along the downward course of the trajectory (Figures 2 and 3) require larger VTAs to reach PVimp. Category 3 spots were balanced between the two hemispheres with a larger volume of distribution in the right hemisphere. The overlap between imp and se volumes in the second implanted hemisphere, which aligns with the transitory lesioning effect, could be due to the colocation of Vim neuron clumps and fiber pathways (Figure 5). These findings also align with pioneering works (38): the imp volume of the first implanted hemisphere corresponds to the camptotomy site; the overlay of imp and se volumes of the second implanted hemisphere corresponds to both the thalamo-capsular and the relay-nuclei sites. The volumes of effects fit with the related structures and circuits of the ET syndrome (Figure 6) (63–66). The left imp volume is clearly related to the cortico-ponto-cerebello-rubro-thalamic pathway, whereas the right imp-se volume is more related to the cortico-thalamic and thalamo-cortical systems. Left imp volume, and right at a lesser extent, are also related to the cortico-subthalamic fibers (Figures 5, 6 and Supplementary Figure S2).

**Figure 6:**
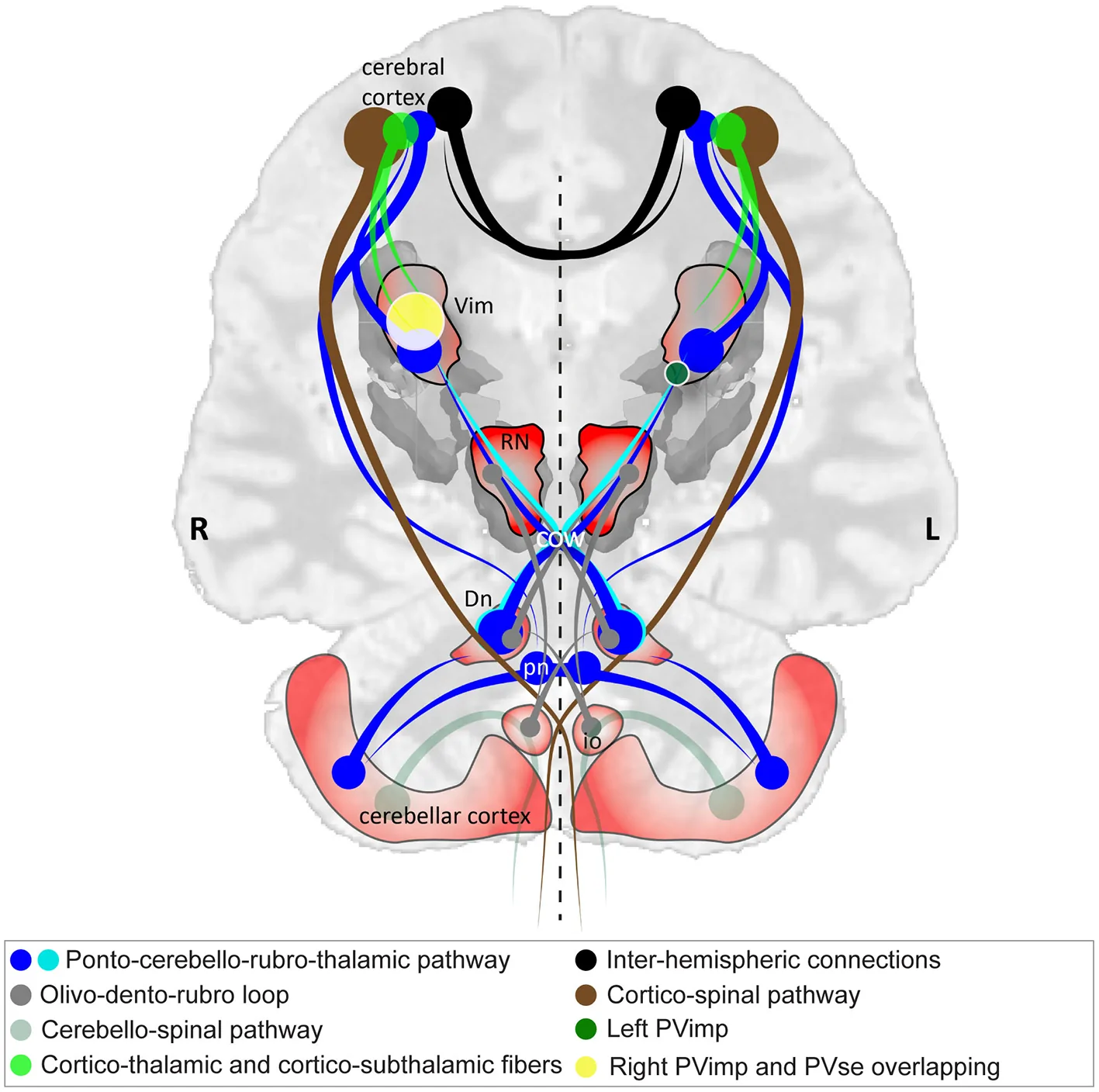
Related circuitry of ET syndrome: (1) main dysregulated structures, cerebellar cortex, dentate nucleus (Dn), inferior olivary (io), red nucleus (RN) and Vim; (2) main related circuits, cortico-ponto-cerebello-rubro-thalamic pathway (decussating, dark blue, and non-decussating, pale blue; pn, pontine nuclei), olivo-dento-rubro loop (Guillain Mollaret triangle; gray), cerebello-spinal pathway (faded dark green); (3) other related systems and structures, cortico-thalamic and cortico-subthalamic fibers (light green), inter-hemispheric connections (black), commissure of Wernekink (cow), cortico-spinal pathway (brown); (4) right (R) and left (L) volumes of effects (colored circles), left PVimp (dark green), right PVse and PVimp overlapping (yellow); midline, vertical dashed line.

Our findings reveal a transitory, tangible, lesioning effect (14, 36) following the first DBS lead implantation, and this a priori irrespective of the hemisphere. Determining whether initial lesioning of the right or left hemisphere leads to distinct functional outcomes remains challenging. Studies provide avenues for further reflection, such as laterality of vestibular system (67), and asymmetry of effects (15, 68–71). In retrospective or routine clinical settings, such as our series, these differences are minimal and result in largely equivalent, mirrored outcomes. To truly differentiate the effects of initiating implantation in the right vs. the left hemisphere, one must identify clear, lateralized functional markers across an extended circuitry (Figure 6) while accounting for ipsilateral engagement. Ipsilateral effect is largely supported by the presence of several cortical and subcortical systems crossing the midline (Figure 6) (12). Regardless of the underlying mechanism, we found a reduced spatial differentiation between PVimp and PVse in the second implanted hemisphere, corroborated by stimulation spot cluster analysis (Figure 4 and Supplementary Table S3). In fact, inter-hemispheric comparisons revealed that optimal positions were more medial, and inferior in the left hemisphere than in the right (Figures 4, 5), mirroring the asymmetry observed for PVimp. These results suggest that the left lesioning effect achieved a near-complete tremor suppression within a larger, functionally undifferentiated central region of the right Vim, encompassing both anti-tremor and side effect–related areas. The asymmetry of PVs should be minimal in the absence of lesioning effect. Initial exploration and implantation in the naïve hemisphere revealed a confined area of improvement, which widened the contralateral PVimp resulting in an overlap between PVimp and PVse. This expansion likely accounts for the masking of hemisphere-specific lesion effects when data from both hemispheres are averaged. Moreover, in our series, the clinical effect on the left hemibody, should be consecutive to the addition of high frequency stimulation of the right semi-microelectrode with a concomitant transitory ipsilesioning effect of the left DBS lead. Ipsilateral effect is largely supported by the presence of several cortical and subcortical systems crossing the midline (Figure 6) (12). Thus, taken as a whole, the findings shed light on the still undeciphered dual-hemispheric controls of the pathologic tremor circuit in ET syndrome (72–74).

### 4.2 Strengths and limitations

The overall interpretation should be weighted by the retrospective design and the limited sample size of data. Although full data were collected in a consecutive series of patients, our findings would benefit of further prospective works. However, the impact of the limited sample size is mitigated by the high number of stimulation spots per patient and by the use of a Bayesian *t*-test, which is more robust to outliers than conventional methods such as the Wilcoxon test and has been shown to provide reliable estimates in relatively small samples (50, 75). Another methodological consideration is spatial normalization to a common anatomical space. We used individualized-VTA further grouped (non-linear registration) in a group-specific MRI template enabling analysis of effects, registered (rigid) to the DB-MA atlas. As in other studies using space normalization, such as driving-force VTAs, normalized individual and group datasets [non-linear registration to the DISTAL atlas (MNI space) (76), or the Ilinsky atlas (77)], as well as individualized, semi-supervised, diffusion-based VTAs [interpreted according to the Morel's atlas segmentations (78)], the topographic analysis may have been affected by the normalization procedure. DTI-derived anisotropic conductivity models may improve the estimation of VTA shape and extent, potentially refining the localization of clinically relevant regions (79–81). A notable strength of this study is the reliance on intra-operative data, which provides a denser sampling of stimulation spots along the explored trajectories compared with postoperative assessments based on DBS lead contacts (larger diameter than semi-microelectrodes and constrained to fixed discrete positions). Future prospective studies with standardized stimulation protocols and randomized first hemisphere implantation could further investigate hemispheric differences and the anti-tremor specificity of the identified regions. Notably, while the categorization of stimulation spots ensured consistent and reproducible interhemispheric comparisons, it may have influenced the observed hemispheric differences.

## 5 Conclusion

This study integrated probabilistic mapping of stimulation effects with a systematic evaluation of stimulation spots categorized according to clinical criteria – based on the therapeutic window and side effect occurrence. The findings enhance the understanding of the neuro-architectural correlates of anti-tremor benefit observed intraoperatively, notably regarding the lesioning effect. Although we cannot conclude definitively on the precise modulation of the dysfunctional circuit in ET, the findings could be translated into further applicable concepts, supporting more effective programming strategies. First of all, our results point out the interest to decouple the first and second implanted hemisphere in the analysis of acute intraoperative effects. In addition, the intraoperative transitory lesioning effect highlights the functional rearrangement in the contralateral thalamus, leading to a larger spread, with a slight topographic shift, of clinically relevant positions. The large PV in the second explored hemisphere should be considered when positioning the DBS lead, given that the deep contacts could be effective, mirroring the PVimp of the first implanted hemisphere once the transitory lesioning effect wears off. Nevertheless, this might be leveraged during chronic stimulation, where both DBS electrodes exert contra- and ipsilateral effects, that should include the geometric and functional modifications of stimulation effects. A possible development could be the application of novel chronic DBS strategies, such as interleaved interhemispheric multi-energy and -contact stimulations. In this approach, stimulation intensity would alternate between hemispheres, with one hemisphere receiving a higher (conventional therapeutic) amplitude while the contralateral hemisphere simultaneously receives a lower amplitude, according to different contacts, followed by periodic switching of intensity levels. Future works on larger patient cohorts, combined with the integration of quantitative improvement scores into position categorization, could help to refine this framework to diverse patient populations and movement disorders, such as motor pathologies, e.g., Parkinson's disease and dystonia, and non-motor, such as obsessive-compulsive disorders, with clinically relevant volumes defined according to the specific symptoms being targeted.

## Data Availability

The raw data supporting the conclusions of this article will be made available by the authors upon request, without undue reservation.

## Footnotes

1 https://liu.se/en/article/ne-downloads.
