# Supplementary material for: Intraoperative asymmetry of thalamic stimulation mapping in Essential Tremor: specificity of first hemisphere implantation and transitory lesioning effect
Source: Front Neurol. 2026 Oct 15;17:1893911. doi: 10.3389/fneur.2026.1893911 (PMC13716563; doi:10.3389/fneur.2026.1893911)
Supplement: Supplementary file 1 [file Supplementary_file_1.docx]

Supplementary Material

*Vittoria Bucciarelli, Dorian Vogel, Jérôme Coste, Rémi Chaix, Simone Hemm and Jean-Jacques Lemaire*

# 1 VTA modelling

Patient-specific Volume of Tissue Activated (VTA) modelling was performed to estimate the spatial extent of tissue influenced by each stimulation condition. VTAs were generated in the native anatomical space of each patient based on the individual electrode localization and stimulation parameters recorded during intraoperative testing. Subsequently, individual VTAs were transformed into a common anatomical space to enable cohort-level spatial analyses and probabilistic mapping.

The computational domain was defined using patient-specific anatomical reconstructions. Electrical conductivities were assigned to the different tissue compartments according to previously reported values for DBS electric field modelling. CSF and blood were set to 2.0 Siemens/meter (S/m) and 0.7 S/m, respectively. Considering the frequency (130 Hz) and pulse length (60 μs) of the stimulation, to gray matter was assigned 0.123 S/m and to white matter 0.075 S/m. The brain tissue was considered an isotropic conductive medium. The conductivity model accounts for the different electrical properties of the tissues surrounding the electrode, which influence the spatial distribution of the generated electric field.

The intraoperative electrode was reconstructed in the patient anatomical space according to the planned implantation trajectory. A model of the stimulating contact of the exploration electrode and the guide tube was developed. The end of the guide tube was fixed 12 mm above the chosen target point, i.e., above the a priori optimal anatomic spot. A second exploration electrode and guide tube model was positioned in parallel at a distance of 2 mm.

A monopolar configuration was conducted using the guide tube as the reference electrode setting it to ground, and the active electrode set to the same current as used during the stimulation tests. The exterior boundaries of the tissue model were set to electrical insulation.

The electric field (EF) distribution generated by each stimulation configuration was estimated by solving the bioelectric volume conductor problem using finite element modelling (FEM). The model assumes quasi-static conditions, where the extracellular electric potential distribution is described by:

$$\nabla\cdot\left( \sigma\nabla\phi\right)=0$$

where $\phi$represents the extracellular electrical potential and $\sigma$represents the electrical conductivity tensor of the surrounding tissues. The mesh density was defined by the built in physics-controlled mesh generator, where the smallest elements (0.204 mm) were located nearby the stimulating contacts in order to capture the strong EF gradients.

The VTA was estimated using an electric field thresholding approach based on the relationship between extracellular electric field magnitude and neural activation described by (Åström et al., 2015). Voxels with an electric field magnitude exceeding 0.2 V/mm were considered part of the estimated VTA. This threshold-based approach provides an approximation of the volume of neural tissue potentially influenced by stimulation and has been widely used for DBS modelling studies.

# 2 Figure S1

Coronal (top row) and sagittal (bottom row) cross-sections of PVimp and PVse (inlay, section positions).

# 3 Tables SM

Table S1. Centroid distances (mm) from structures of DB-MA atlas of Probabilistic Volume of improvement (PVimp) and Probabilistic Volume of side effect (PVse) for left and right hemisphere; inf.=inferior, post.=posterior, med.=medial, lat.=lateral, sup.=superior, ant.=anterior. Abbreviations: Vim: ventrointermediate nucleus of thalamus; ZI: zona incerta; VO: ventrooral nucleus of thalamus; VC: ventrocaudal nucleus of the thalamus; STN: the subthalamic nucleus; F-H-F: field H that contains the prelemniscal radiations; TH-fa: thalamic fascicle

| Volume | Centroids distances from Vim | | | | | Hemisphere |
| --- | --- | --- | --- | --- | --- | --- |
| PVimp | 5.05 inf. | | 1.61 post. | 2.64 med. | | Left |
| PVse | 1.08 inf. | | 2.85 post. | 1.53 lat. | | Left |
| PVimp | 0.75 inf. | | 2.36 post. | 0.13 lat. | | Right |
| PVse | 0.65 inf. | | 2.49 post. | 0.4 lat. | | Right |
|  | **Centroids distances from STN** | | | | | **Hemisphere** |
| PVimp | 4.62 sup. | | 7.25 post. | 0.42 lat. | | Left |
| PVse | 8.59 sup. | | 8.49 post. | 4.59 lat. | | Left |
| PVimp | 9.02 sup. | | 8.03 post. | 2.92 lat. | | Right |
| PVse | 9.12 sup. | | 8.16 post. | 3.19 lat. | | Right |
|  | **Centroids distances from ZI** | | | | | **Hemisphere** |
| PVimp | 2.15 sup. | | 5.49 post. | 0.63 med. | | Left |
| PVse | 6.12 sup. | | 6.73 post. | 3.54 lat. | | Left |
| PVimp | 5.97 sup. | | 6.72 post. | 1.84 lat. | | Right |
| PVse | 6.07 sup. | | 6.85 post. | 2.11 lat. | | Right |
|  | **Centroids distances from F-H-F** | | | | | **Hemisphere** |
| PVimp | 3.16 sup. | 1.97 post. | | | 1.57 lat. | Left |
| PVse | 7.13 sup. | 3.21 post. | | | 5.74 lat. | Left |
| PVimp | 7.92 sup. | 2.7 post. | | | 3.76 lat. | Right |
| PVse | 8.02 sup. | 2.83 post. | | | 4.03 lat. | Right |
|  | **Centroids distances from VO** | | | | | **Hemisphere** |
| PVimp | 4.47 inf. | 5.63 post. | | | 0.02 lat. | Left |
| PVse | 0.5 inf. | 6.87 post. | | | 4.19 lat. | Left |
| PVimp | 0.44 inf. | 6.36 post. | | | 2.21 lat. | Right |
| PVse | 0.34 inf. | 6.49 post. | | | 2.48 lat. | Right |
|  | **Centroids distances from VC** | | | | | **Hemisphere** |
| PVimp | 4.0 inf. | 3.28 ant. | | | 3.02 med. | Left |
| PVse | 0.03 inf. | 2.04 ant. | | | 1.15 lat. | Left |
| PVimp | 0.29 sup. | 2.47 ant. | | | 0.36 med. | Right |
| PVse | 0.39 sup. | 2.34 ant. | | | 0.09 med. | Right |
|  | **Centroids distances from TH-fa** | | | | | **Hemisphere** |
| PVimp | 1.03 inf. | 6.81 post. | | | 0.02 med. | Left |
| PVse | 2.94 sup. | 8.05 post. | | | 4.15 lat. | Left |
| PVimp | 3.26 sup. | 7.38 post. | | | 2.17 lat. | Right |
| PVse | 3.36 sup. | 7.51 post. | | | 2.44 lat. | Right |

Table S2. Centroid distances (mm) from structures of DB-MA atlas of stimulation spots clusters for left and right hemisphere; inf.=inferior, post.=posterior, med.=medial, lat.=lateral, sup.=superior, ant.=anterior. Abbreviations: Vim: ventrointermediate nucleus of thalamus; ZI: zona incerta; VO: ventrooral nucleus of thalamus; VC: ventrocaudal nucleus of the thalamus; STN: the subthalamic nucleus; F-H-F: field H that contains the prelemniscal radiations; TH-fa: thalamic fascicle

| Stim. spot cluster | Centroids distances from Vim | | | Hemisphere |
| --- | --- | --- | --- | --- |
| 2a-1 | 0.5 sup. | 1.23 post. | 3.08 lat. | Left |
| 2a-2 | 4.07 inf. | 2.76 post. | 1.08 med. | Left |
| 2c | 1.02 sup. | 0.87 post. | 1.61 lat. | Left |
| 3 | 1.66 inf. | 1.42 post. | 0.17 med. | Left |
| 2a | 0.38 sup. | 2.67 post. | 0.29 med. | Right |
| 2c-1 | 1.67 sup. | 0.34 ant. | 0.82 lat. | Right |
| 2c-2 | 0.84 sup. | 3.77 ant. | 0.91 lat. | Right |
| 3-1 | 2.78 sup. | 1.68 post. | 0.6 lat. | Right |
| 3-2 | 0.17 inf. | 0.75 ant. | 0.12 lat. | Right |
|  | **Centroids distances from STN** | | | **Hemisphere** |
| 2a-1 | 10.17 sup. | 6.88 post. | 6.14 lat. | Left |
| 2a-2 | 5.6 sup. | 8.41 post. | 1.99 lat. | Left |
| 2c | 10.69 sup. | 6.51 post. | 4.67 lat. | Left |
| 3 | 8.01 sup. | 7.06 post. | 2.9 lat. | Left |
| 2a | 10.15 sup. | 8.34 post. | 2.51 lat. | Right |
| 2c-1 | 11.43 sup. | 5.34 post. | 3.61 lat. | Right |
| 2c-2 | 10.61 sup. | 1.91 post. | 3.71 lat. | Right |
| 3-1 | 12.54 sup. | 7.36 post. | 3.39 lat. | Right |
| 3-2 | 9.59 sup. | 4.93 post. | 2.92 lat | Right |
|  | **Centroids distances from ZI** | | | **Hemisphere** |
| 2a-1 | 7.7 sup. | 5.11 post. | 5.09 lat. | Left |
| 2a-2 | 3.14 sup. | 6.64 post. | 0.94 lat. | Left |
| 2c | 8.23 sup. | 4.75 post. | 3.62 lat. | Left |
| 3 | 5.55 sup. | 5.3 post. | 1.85 lat. | Left |
| 2a | 7.1 sup. | 7.03 post. | 1.43 lat. | Right |
| 2c-1 | 8.39 sup. | 4.03 post. | 2.53 lat. | Right |
| 2c-2 | 7.56 sup. | 0.6 post. | 2.63 lat. | Right |
| 3-1 | 9.49 sup. | 6.04 post. | 2.31 lat. | Right |
| 3-2 | 6.54 sup. | 3.62 post. | 1.84 lat. | Right |
|  | **Centroids distances from F-H-F** | | | **Hemisphere** |
| 2a-1 | 8.71 sup. | 1.59 post. | 7.29 lat. | Left |
| 2a-2 | 4.14 sup. | 3.12 post. | 3.13 lat. | Left |
| 2c | 9.23 sup. | 1.23 post. | 5.81 lat. | Left |
| 3 | 6.55 sup. | 1.78 post. | 4.04 lat. | Left |
| 2a | 9.05 sup. | 3.01 post. | 3.35 lat. | Right |
| 2c-1 | 10.34 sup. | 0.01 post. | 4.45 lat. | Right |
| 2c-2 | 9.51 sup. | 3.42 ant. | 4.55 lat. | Right |
| 3-1 | 11.44 sup. | 2.03 post. | 4.23 lat. | Right |
| 3-2 | 8.49 sup. | 0.4 ant. | 3.76 lat. | Right |
|  | **Centroids distances from VO** | | | **Hemisphere** |
| 2a-1 | 1.08 sup. | 5.25 post. | 5.74 lat. | Left |
| 2a-2 | 3.48 inf. | 6.78 post. | 1.58 lat. | Left |
| 2c | 1.61 sup. | 4.89 post. | 4.27 lat. | Left |
| 3 | 1.07 inf. | 5.44 post. | 2.49 lat. | Left |
| 2a | 0.69 sup. | 6.67 post. | 1.79 lat. | Right |
| 2c-1 | 1.98 sup. | 3.67 post. | 2.9 lat. | Right |
| 2c-2 | 1.15 sup. | 0.24 post. | 2.99 lat. | Right |
| 3-1 | 3.08 sup. | 5.68 post. | 2.68 lat. | Right |
| 3-2 | 0.14 sup. | 3.26 post. | 2.2 lat. | Right |
|  | **Centroids distances from VC** | | | **Hemisphere** |
| 2a-1 | 1.54 sup. | 3.66 ant. | 2.7 lat. | Left |
| 2a-2 | 3.02 inf. | 2.13 ant. | 1.45 med. | Left |
| 2c | 2.07 sup. | 4.02 ant. | 1.23 lat. | Left |
| 3 | 0.61 inf. | 3.47 ant. | 0.54 med. | Left |
| 2a | 1.42 sup. | 2.16 ant. | 0.78 med. | Right |
| 2c-1 | 2.7 sup. | 5.16 ant. | 0.33 lat. | Right |
| 2c-2 | 1.88 sup. | 8.59 ant. | 0.42 lat. | Right |
| 3-1 | 3.81 sup. | 3.15 ant. | 0.11 lat. | Right |
| 3-2 | 0.86 sup. | 5.57 ant. | 0.37 med. | Right |
|  | **Centroids distances from TH-fa** | | | **Hemisphere** |
| 2a-1 | 4.52 sup. | 6.43 post. | 5.7 lat. | Left |
| 2a-2 | 0.05 inf. | 7.96 post. | 1.54 lat. | Left |
| 2c | 5.04 sup. | 6.07 post. | 4.23 lat. | Left |
| 3 | 2.36 sup. | 6.61 post. | 2.45 lat. | Left |
| 2a | 4.39 sup. | 7.69 post. | 1.75 lat. | Right |
| 2c-1 | 5.68 sup. | 4.69 post. | 2.86 lat. | Right |
| 2c-2 | 4.85 sup. | 1.26 post. | 2.95 lat. | Right |
| 3-1 | 6.78 sup. | 6.7 post. | 2.64 lat. | Right |
| 3-2 | 3.84 sup. | 4.28 post. | 2.16 lat. | Right |

**Table S3***.* Number and percentage of stimulation spots located within the probabilistic volumes associated with clinical improvement (PVimp) and side effects (PVse), stratified by stimulation category and hemisphere. Percentages represent the proportion of spots within each category falling inside the corresponding PV

| Category | Spots in PVimp [%] | Spots in PVse [%] |
| --- | --- | --- |
| Left hemisphere | | |
| 1a | 2 [11.7%] | 10 [58.8%] |
| 1b | 1 [2.6%] | 7 [18.4%] |
| 2a | 6 [14.3%] | 34 [80.9%] |
| 2b | 1 [20%] | 5 [100%] |
| 2c | 4 [2.6%] | 88 [56.7%] |
| 3 | 10 [13.3%] | 62 [82.6%] |
| Right hemisphere | | |
| 1a | 23 [69.7%] | 25 [75.8%] |
| 1b | 2 [10%] | 2 [10%] |
| 2a | 18 [75%] | 18 [75%] |
| 2b | 13 [72.2%] | 11 [61.1%] |
| 2c | 32 [36%] | 39 [43.8%] |
| 3 | 49 [55.1%] | 47 [52.8%] |

# 4 Figure S2: Architecture of Vim area

DB-MA (Lemaire et al., 2024) atlas registration; top row, lateral (left), anterior (right) 3D views of sections going through the centre of Vim (acpc alignment; mic, intercommissural coronal plane; mid, midline sagittal plane); bottom row, same sections, with contours of structures on related fiber systems (1-mm thickness slice; fiber tracings in gray; Diffusion Color Encoded map); RN, red nucleus, stf, subthalamic tegmental fields, *F-H-F, (1) rubro-thalamo-cortical fibers, (2) trans-zona-cortico-subthalamic fibers, (3) thalamo-cortical fibers, (4) subthalamo-cortical fibers, (5) VC-cortical fibers (see main text for abbreviations).

# 5 References

Åström, M., Diczfalusy, E., Martens, H., and Wårdell, K. (2015). Relationship between Neural Activation and Electric Field Distribution during Deep Brain Stimulation. *IEEE Trans. Biomed. Eng.* 62, 664–672. doi: 10.1109/TBME.2014.2363494

Lemaire, J.-J., Chaix, R., Dautkulova, A., Sontheimer, A., Coste, J., Marques, A.-R., et al. (2024). An MRI Deep Brain Adult Template With An Advanced Atlas-Based Tool For Diffusion Tensor Imaging Analysis. *Sci. Data* 11, 1189. doi: 10.1038/s41597-024-04053-x
